# Relationship between Brain Tissue Changes and Blood Biomarkers of Cyclophilin A, Heme Oxygenase-1, and Inositol-Requiring Enzyme 1 in Patients with Alzheimer’s Disease

**DOI:** 10.3390/diagnostics11050740

**Published:** 2021-04-21

**Authors:** Hyon-Il Choi, Kiyoon Kim, Jiyoon Lee, Yunjung Chang, Hak Young Rhee, Soonchan Park, Woo-In Lee, Wonchae Choe, Chang-Woo Ryu, Geon-Ho Jahng

**Affiliations:** 1 Department of Radiology, Kyung Hee University Hospital at Gangdong, 892 Dongnam-ro, Gangdong-Gu, Seoul 05278, Korea; rad.hyeonil@gmail.com (H.-I.C.); parkkhmc96@hanmail.net (S.P.); 2 Department of Biochemistry and Molecular Biology, Graduate School, Kyung Hee University, 26 Kyung Hee Dae-ro, Dongdaemun-gu, Seoul 02447, Korea; soowonsky@gmail.com; 3 Department of Biomedical Engineering, Undergraduate School, College of Electronics and Information, Kyung Hee University, 1732 Deogyeong-daero, Giheung-gu, Yongin-si, Seoul 17104, Korea; little8867@naver.com (J.L.); ynjngchang@gmail.com (Y.C.); 4 Department of Medicine, College of Medicine, Kyung Hee University, 26 Kyung Hee Dae-ro, Dongdaemun-gu, Seoul 02447, Korea; azzo73@gmail.com (H.Y.R.); wileemd@khu.ac.kr (W.-I.L.); 5 Department of Neurology, Kyung Hee University Hospital at Gangdong, 892 Dongnam-ro, Gangdong-gu, Seoul 05278, Korea; 6 Department of Laboratory Medicine, Kyung Hee University Hospital at Gangdong, 892 Dongnam-ro, Gangdong-gu, Seoul 05278, Korea; 7 Department of Biochemistry and Molecular Biology, College of Medicine, Kyung Hee University, 26 Kyung Hee Dae-ro, Dongdaemun-gu, Seoul 02447, Korea; wchoe@khu.ac.kr

**Keywords:** Alzheimer′s disease, gray matter volume, blood-based biomarker, heme oxygenase-1, inositol-requiring enzyme 1, cyclophilin A

## Abstract

Cyclophilin A (CypA), heme oxygenase-1 (HO-1), and inositol-requiring enzyme 1 (IRE1) are believed to be associated with Alzheimer’s disease (AD). In this study, we investigated the association between gray matter volume (GMV) changes and blood levels of CypA, HO-1, and IRE1 in cognitively normal (CN) subjects and those with amnestic mild cognitive impairment (aMCI) and AD. Forty-five elderly CN, 34 aMCI, and 39 AD subjects were enrolled in this study. The results of voxel-based multiple regression analysis showed that blood levels of CypA, HO-1, and IRE1 were correlated with GMV on brain magnetic resonance imaging (MRI) in the entire population (*p* = 0.0005). The three serum protein levels were correlated with GMV of signature AD regions in the population as a whole. CypA values increased with increasing GMV in the occipital gyrus (*r* = 0.387, *p* < 0.0001) and posterior cingulate (*r* = 0.196, *p* = 0.034). HO-1 values increased with increasing GMV at the uncus (*r* = 0.307, *p* = 0.0008), lateral globus pallidus and putamen (*r* = 0.287, *p* = 0.002), and hippocampus (*r* = 0.197, *p* = 0.034). IRE1 values decreased with increasing GMV at the uncus (*r* = −0.239, *p* = 0.010) and lateral globus pallidus and putamen (*r* = −0.335, *p* = 0.0002). Associations between the three serum protein levels and regional GMV indicate that the blood levels of these biomarkers may reflect the pathological mechanism of AD in the brain.

## 1. Introduction

Alzheimer′s disease (AD), the most common cause of dementia, is considered an inevitable consequence of aging that is exacerbated by a genetic predisposition. AD is characterized by the accumulation of amyloid-β plaques outside cells and around neurons and the strings of neurofibrillary tangles of tau protein inside cells [1]. AD typically destroys neurons and their connections in the parts of the brain involved in memory. Amnestic mild cognitive impairment (aMCI) is a condition in which memory problems are more severe than in normal elderly, but not serious enough to affect daily life. aMCI is a risk factor for developing AD and is considered an interim phase of AD [2].

Many researchers have focused on the identification of blood-borne biomarkers for AD, and amyloid-beta protein has been targeted [3,4]. However, until now, a blood-borne biomarker with a direct association to the pathogenesis of AD has not been found. Accumulating evidence suggests that reactive oxygen species (ROS) generation and endoplasmic reticulum (ER) stress could increase the risk of developing AD [5,6,7]. Three proteins in the plasma are associated with neurotoxicity induced by ER stress and oxidative damage: cyclophilin A (CypA), heme oxygenase-1 (HO-1), and inositol-requiring enzyme 1 (IRE1). These three proteins thus may have an association with AD pathogenesis and are potential blood-borne biomarkers. First, CypA, also known as peptidylprolyl isomerase A (PPIA), has been shown to play a regulatory role in the maintenance of blood–brain barrier (BBB) integrity in the central nervous system via a complex with nuclear factor κB and matrix metalloproteinase 9 under control of apolipoprotein E (APOE). However, it can mediate BBB dysfunction in the presence of APOE4, a major genetic risk factor for AD [8,9]. Recent studies have suggested that the breakdown of the BBB is an early sign of human cognitive dysfunction, including AD [9]. Second, HO-1, which is responsible for the degradation of heme to biliverdin/bilirubin, is associated with cellular oxidative stress, which plays a role in neurofibrillary tangles [10]. Glial HO-1 expression may also impact cell survival and neuroplasticity in AD by modulating brain sterol/oxysterol metabolism and the degradation of tau by the proteasome [11,12]. Finally, IRE1 is a major stress transducer in ER stress and abnormal protein aggregation, prominent in the unfolded protein response (UPR), which reflects disturbed homeostasis in the ER [13]. An experimental model showed that UPR induction prevents iron accumulation and oligodendrocyte loss, which are shown in AD pathology [14].

Volumetric brain magnetic resonance imaging (MRI) is an effective tool in the assessment of brain tissue atrophy in AD. Several studies have reported that prominent medial temporal atrophy is typically present in AD and can be used as an imaging biomarker and sentinel sign of neurodegeneration in AD [15]. In aMCI, atrophy is noticeably restricted to the medial temporal lobe [16]. Hence, neuroimaging is a useful tool for characterizing the severity of cognitive impairment and the early identification of aMCI and AD. However, there are limitations in their application as a single modality.

One potential strategy to improve the use of fluid biomarkers of brain injury in AD is by analyzing them in parallel with neuroimaging. Studies have investigated the relationship between structural changes of the brain and cerebrospinal fluid (CSF) biomarkers in AD and aMCI [17], microglial activation in aMCI [18], and plasma amyloid-ß oligomerization in AD and aMCI [19]. However, few studies have explored the relationship between certain blood biomarkers and brain tissue volume losses in AD and aMCI. Assessing circulating proteins HO-1, CypA, and IRE1 with combined neuroimaging to elucidate the mechanisms of neuronal damage in AD may help validate the continued use of these proteins in AD research. However, to our knowledge, no study has evaluated the relationship between brain structural changes and blood levels of these proteins. Therefore, we hypothesized that neuronal structural changes in AD/aMCI may be associated with blood levels of either CypA, HO-1, or IRE, or in combination. In this study, we evaluated the relationship between brain tissue volume changes as imaging biomarkers and CypA, HO-1, and IRE1 as blood biomarkers in cognitively normal (CN) elderly, aMCI, and AD participants.

## 2. Results

### 2.1. Participant Characteristics

Age was significantly different between the CN and other groups (F = 20.697, *p* < 0.001). Sex was not significantly different among the three groups (χ^2^ = 3.741, *p* = 0.154) and females predominated each subject group. The Korean (K)-Mini-Mental State Examination (MMSE) scores were significantly different between the three subject groups (F = 65.904, *p* < 0.001), as expected. The AD group had fewer years of education than the CN group (F = 4.051, *p* = 0.020) (Table 1).

Global GMV was significantly different between AD and other groups and decreased with increasing disease severity. Global white matter volume (WMV) was significantly different between AD and CN groups and also decreased with increasing disease severity. Global CSF volume was significantly different among the three groups (F = 39.614, *p* < 0.001), showing a positive correlation with disease severity. Total intracranial volume (TIV) was not significantly different among the three groups (F = 0.505, *p* = 0.605) (Table 1).

Comparisons of CypA, HO-1, and IRE1 blood levels showed that CypA (F = 3.089, *p* = 0.049) and HO-1 (F = 3.415, *p* = 0.036) values were significantly different among the three subject groups, but these results were not significant on post hoc test. IRE1 (F = 2.995, *p* = 0.054) values were similar among the three subject groups (Table 1).

### 2.2. Correlation Analyses among Subject Characteristics and Blood Biomarkers

**Figure 1:**
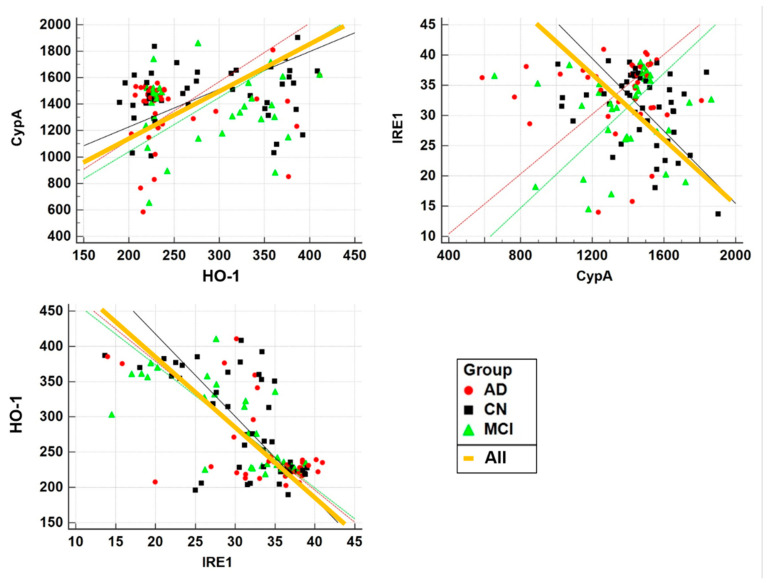
Pearson correlation analyses of the levels of the three blood-based biomarkers in all participants and in subgroups. CypA, cyclophilin A; HO-1, heme oxygenase-1; IRE1, inositol-requiring enzyme 1; CN, cognitive normal; MCI, amnestic mild cognitive impairment; AD, Alzheimer’s disease.

**Figure 2:**
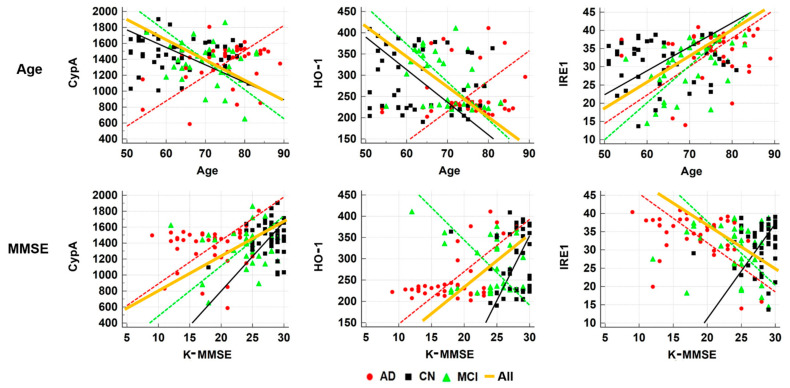
Correlation analyses between levels of the three blood-based biomarkers and age or MMSE score in all participants and in subgroups. MMSE, Mini-Mental State Examination Score; CypA, cyclophilin A; HO-1, heme oxygenase-1; IRE1, inositol-requiring enzyme 1; CN, cognitive normal; MCI, amnestic mild cognitive impairment; AD, Alzheimer’s disease.

CypA level was not significantly correlated with levels of HO-1 and IRE1, but HO-1 level was significantly negatively correlated with IRE1 in the population as a whole (*r* = −0.6639, *p* < 0.0001) and each group (Table 2, Figure 1). The CypA value was significantly positively correlated with MMSE score (*r* = 0.2154, *p* = 0.0192) and global GMV (*r* = 0.2197, *p* = 0.0168). HO-1 was significantly negatively correlated with age (*r* = −0.2262, *p* = 0.0138), but was positively correlated with K-MMSE score (*r* = 0.2495, *p* = 0.0064) and with global GMV (*r* = 0.1925, *p* = 0.0368). IRE1 was significantly positively correlated with age (*r* = 0.2220, *p* = 0.0157), but was negatively correlated with K-MMSE score (*r* = −0.2411, *p* = 0.0085) and global GMV (*r* = −0.2125, *p* = 0.0209) (Table 2, Figure 2). Partial correlation after adjusting for age revealed only a significant relationship between CypA and MMSE score.

### 2.3. Voxel-Based Multiple Regression Analyses

**Figure 3:**
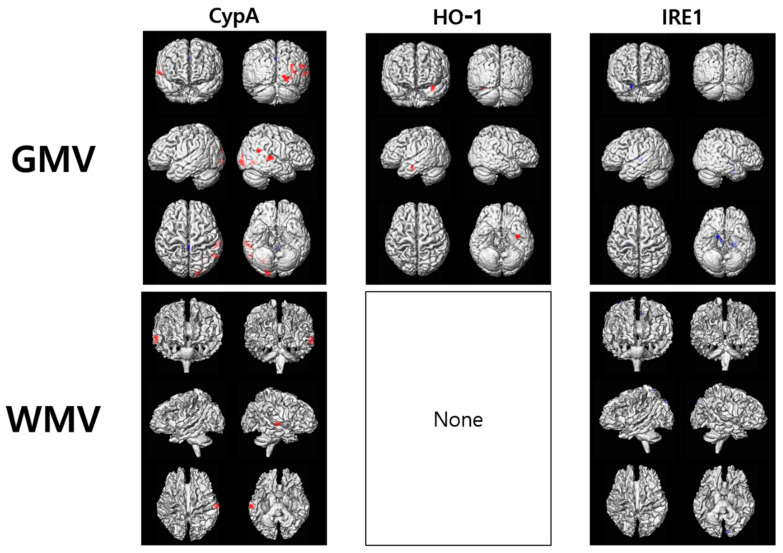
Voxel-based multiple regression analyses between brain tissue volumes and the levels of the CypA, HO-1, and IRE1. CypA, cyclophilin A; HO-1, heme oxygenase-1; IRE1, inositol-requiring enzyme 1. The red and blue colors indicate positive and negative associations, respectively.

GMV was positively correlated with CypA and HO-1 but negatively correlated with IRE1. CypA values were increased with increasing GMV in the occipital lobe (Cluster 1), temporal lobe, and cerebellum. HO-1 values increased with increasing GMV in the uncus (Cluster 2) and postcentral gyrus. IRE1 values decreased with increasing GMV in the left lateral globus pallidus and putamen (Cluster 3) and hypothalamus (Figure 3, Supplementary Table S1).

WMV was positively correlated with CypA but negatively correlated with IRE1. CypA values increased with increasing WMV in the inferior parietal lobule and the middle temporal gyrus (Cluster 4). IRE1 values decreased with increasing WMV in the precuneus and cuneus (Cluster 5) (Figure 3, Supplementary Table S2). These cluster areas were defined as regions of interest (ROIs) to obtain GMV or WMV and in the following ROI-based analyses.

### 2.4. ROI-Based Correlation Analyses

**Figure 4:**
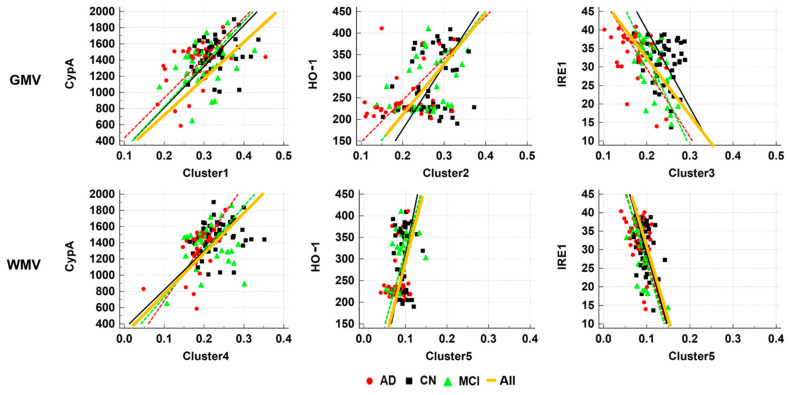
Pearson correlation analyses between the three blood-based biomarkers and brain tissue volumes in all participants and in subgroups. GMV, gray matter volume; WMV, white matter volume; cyclophilin A, CyPA; HO-1, heme oxygenase-1; IRE1, inositol-requiring enzyme 1; CN, cognitive normal; MCI, amnestic mild cognitive impairment; AD, Alzheimer’s disease.

First, CypA was positively correlated with GMV at Cluster 1 (*r* = 0.387, *p* < 0.0001), Cluster 4 (*r* = 0.223, *p* = 0.016), and in the posterior cingulate (*r* = 0.196, *p* = 0.034), as well as with WMV at Cluster 4 (*r* = 0.369, *p* < 0.0001). Second, HO-1 was positively correlated with GMV at Cluster 2 (*r* = 0.307, *p* = 0.0008), Cluster 3 (*r* = 0.287, *p* = 0.002), and in the hippocampus (*r* = 0.197, *p* = 0.034), as well as with WMV at Cluster 5 (*r* = 0.225, *p* = 0.015). Finally, IRE1 was negatively correlated with GMV at Cluster 2 (*r* = −0.239, *p* = 0.01), Cluster 3 (*r* = −0.335, *p* < 0.0002), and Cluster 5 (*r* = −0.405, *p* < 0.0001) (Table 3, Figure 4).

## 3. Discussion

In this study, we evaluated the expression of three proteins, CypA, HO-1, and IRE1, in peripheral blood, and investigated the association of these protein levels with GMV changes in CN, aMCI, and AD participants. We found three important results. First, blood levels of HO-1 and IRE1 were significantly correlated with age. Second, blood levels of CypA, HO-1, and IRE1 were significantly correlated with cognitive function. Finally, volumes in some brain areas were positively associated with blood levels of CypA and HO-1 but negatively associated with blood levels of IRE1. These findings suggest that the blood levels of the three proteins are likely indicators of neurodegenerative changes in the brain.

### 3.1. Association of CypA (PPIA) Blood Level

Blood CypA protein levels were lower in AD patients than in CN subjects, but this difference was not statistically significant after post hoc correction. Blood CypA levels decreased with decreasing cognitive function and increased with increasing brain tissue volume. CypA mediates BBB dysfunction in the presence of APOE4 [20]. Even though the contribution of CypA to AD has been recognized, only a few ex vivo animal experiments indicated that the overexpression of CypA is a genetic risk factor of AD. A recent human study found that CSF CypA level was upregulated in a cognitively impaired APOE4 carrier, with increased permeability in the hippocampus and parahippocampal gyrus [21]. Unfortunately, the detailed molecular mechanism of CypA regulation in the blood has not been identified in AD pathology. No previous studies have considered blood CypA level in cognitive function, and there is a lack of evidence to explain why CypA level in peripheral blood in AD varies from the associations seen in brain tissue or CSF. CypA is considered to be a survival or anti-apoptotic factor that protects neuronal cells from oxidative stress. Thus, our data suggest that reduced protection from oxidative damage might be due to decreased levels of plasma CypA in AD patients. However, further research is necessary to establish a direct relationship between blood CypA and cognitive function.

Although the mechanism involving CypA blood level in AD pathophysiology still needs to be elucidated, the results of serum CypA correlation with global/regional GMV supports the usefulness of blood CypA level for identifying AD or aMCI among the elderly. We found a regional correlation with CypA in the right middle occipital gyrus and lingual gyrus (Cluster 1), middle temporal gyrus (Cluster 4), and posterior cingulate. Among these, the middle temporal gyrus primarily accumulates tau protein and shows neurofibrillary tangles in the early stages of AD [20,22]. The posterior cingulate is also a signature region of early volume loss in AD [21]. This relationship between brain tissue and blood CypA suggests that decreased levels of blood CypA are linked pathophysiologically to the eloquent region of cognitive impairment in AD/aMCI. The present study was the first to analyze differences in peripheral CypA depending on the presence of AD/aMCI, and the potential value of plasma CypA level as a blood biomarker for AD should be further investigated.

### 3.2. Association of HO-1 Blood Level

Plasma HO-1 protein levels appeared lower in AD patients than in CN subjects in this study, but this difference was not statistically significant after post hoc correction. Plasma HO-1 protein levels decreased with increasing age, decreasing MMSE score, and decreasing brain tissue volume. HO-1 is implicated in iron accumulation in the brain during normal age-related increases in ferritin [22]. However, it is also thought to be responsible for pathologic iron mobilization and attendant oxidative injury in neurodegenerative diseases including AD [23]. There has been some evidence presented supporting HO-1 as a potential biomarker for aMCI and AD. However, changes of HO-1 in plasma from AD patients remain controversial. In previous studies using human/animal specimens, HO-1 was markedly overexpressed in neurons and astrocytes of the cerebral cortex and hippocampus in AD patients. This overexpression may be induced by oxidative stress provoked by pro-inflammatory cytokines and amyloid burden [24]. In contrast, HO-1 expression was found to be suppressed in the blood of AD patients in other studies [23,25], consistent with our data. Decreased blood HO-1 levels in AD may be caused by accelerated degradation of HO-1 due to an abnormal redox environment. This discrepancy between peripheral blood and brain tissue HO-1 levels was explained in related research indicating the activation of an HO-1 suppressor in AD and aMCI, based on negative correlations of serum HO-1 suppressor levels with MMSE scores. The under-expression of plasma HO-1 can be explained by the hypothesis that HO-1-mediated iron mobilization in AD astrocytes induces the activation of an HO-1 suppressor as a defense mechanism and the HO-1 suppressor may suppress the gene encoding HO-1 in AD peripheral tissues [26]. Altogether, these results suggest that redox stress is an important factor in AD pathology. A (GT)n repeat in the human HO-1 gene promoter region is highly polymorphic, although no particular alleles are associated with AD [27]. In addition, in a Japanese population, no genetic association was found between polymorphisms of HO-1 and Alzheimer’s disease [28].

We observed serum HO-1 levels linked to brain structure through regression analysis with regional GMV including the limbic lobe (Cluster 2), lateral globus pallidus and putamen (Cluster 3), parietal cortex, and hippocampus. These HO-1-responsive areas are reputed to be major signature regions of AD with significant atrophy on progression of cognitive function regardless of age [21,29,30,31]. Therefore, our results suggest that serum HO-1 activity is associated with disease severity and has an early indication in the vulnerable structures of AD. Our results also emphasize the diagnostic value of serum HO-1 through correlation with imaging biomarkers.

### 3.3. Association of IRE1 Plasma Level

The results of this study showed that the behavior of IRE1 was opposite to that of CypA and HO-1. Plasma IRE1 protein levels were higher in AD patients than in CN subjects, but this difference was not statistically significant. Plasma IRE1 protein levels increased with increasing age, increased with decreased cognitive function, and decreased with increasing brain tissue volume. The ER is the main subcellular compartment involved in protein folding and secretion, in addition to lipid synthesis and calcium storage. Pathologic changes in AD, such as the presence of amyloid plaques, neurofibrillary tangles, and oxidative damage, is strongly associated with ER stress [17,32]. IRE1 is a membrane-bound protein and a major stress transducer mediating both adaptive and proapoptotic programs under ER stress. IRE1′s importance as a biomarker and target for the regulation of disease is well established. Previous studies have indicated a correlation between IRE1 activation with the severity of AD neuropathology in ex vivo human specimens and an animal model [33]. However, no previous research has directly measured blood IRE1 level in AD or aMCI patients in vivo. The relationship of IRE1 with regional volume loss suggests the possibility of IRE1 as a blood biomarker of AD and further studies should investigate why serum IRE1 decreases in response to neuronal damage in AD.

In this study, serum IRE1 level was negatively correlated with GMV of the limbic lobe (Cluster 1), globus pallidus and uncus (Cluster 3), and WMV of the cuneus and precuneus (Cluster 5). A regional decrease in WMV was also shown in the middle temporal gyrus associated with HO-1. The WMV reductions in some of these regions, such as the middle temporal gyrus and parietal lobe, were in line with previous MRI studies [34,35], indicating that WMV, in addition to GMV, is an important volume change in AD and aMCI. These findings support the pathophysiologic hypothesis that ER stress correlates with progression in cognitive impairment in AD and aMCI.

### 3.4. Neuroimaging with Plasma Biomarkers

Unfortunately, owing to a lack of relevant studies, the results of this study are unsupported. As described above, apart from HO-1, there have been no major studies on blood levels of CypA or IRE1 related to neurodegenerative changes due to AD and age; therefore, additional studies are needed to clarify the findings of this study. Tau protein or amyloid-β, a byproduct generated from abnormal metabolism in AD, has been studied as a candidate plasma-level biomarker. The three proteins evaluated in this study act as a trigger of neuronal damage through dysfunction of a metabolic circuit in AD, and have potential as diagnostic biomarkers. Because the role of CypA, HO-1, and IRE1 in AD has only been evaluated recently, most research involved ex vivo studies using brain tissue or animal models, and blood levels of these proteins in neurological diseases have not been established. The present study is the first to evaluate these three proteins at the tissue level as blood-borne biomarkers. The results of this study contribute to the growing body of human evidence that suggests structural changes in neuroimaging result from three different mechanisms. CypA, HO-1, and IRE1 are implicated in different mechanisms of brain injury: oxidative damage, BBB breakage, and ER stress, respectively.

These findings provided robust evidence for measuring concurrent variations in both brain imaging and blood biomarkers within an individual, giving greater insight into how these factors are inter-related in AD and aMCI. The use of circulating biomarker expression together with imaging biomarkers as combinatorial predictive biomarkers may improve the timeliness and accuracy of diagnosis of early-stage AD. The identification of elderly individuals at risk for progression of cognition impairment will help in the future design of disease-modifying treatment trials and personalized medicine strategies. The strengths of this study include the fact that protein levels, cognitive ability, and structural brain variables were measured in the same individuals at approximately the same time. This study design using regional brain injury and in vivo neuroimaging is expected to be applied in future studies to assess peripheral biomarkers for neurodegenerative disease. The main findings of this study were the correlation between blood biomarkers and brain tissue changes. However, it is important to note that this correlation was significant only when the whole population, CN included, was considered, whereas the significance disappeared when only patients with cognitive dysfunction were included. Therefore, further studies are needed to validate our results in much larger populations of patients with AD.

### 3.5. Limitations

There were several limitations in this study. First, age was significantly different between groups. This may act as a potential bias in the comparison of serum proteins between groups or in correlation analysis. Although there has not been any published research showing the relationship between the blood level of these three proteins and aging, the expression of these proteins at the tissue level could be associated with aging. To exclude this bias, age was adjusted in voxel-based analysis as a covariate in the general linear model and correlation analyses. In this study, the correlation between MMSE and each protein showed different results according to age adjustment. A further study with a larger population of CN elderly subjects is needed to substantiate the correlation between cognitive function and each of these blood proteins.

Second, the three proteins in this study are not specific to AD but can reflect various systemic diseases or tissue functions. Over-/under-expression of HO-1 levels may be related to inflammation, infection, or metabolic syndrome [36], and that of CypA may be related to vasculopathy or neoplasm [37]. Therefore, it is possible that other underlying conditions that we were not aware of may have affected the results. Although we excluded patients with moderate chronic disease from participation, unknown factors could have affected the blood level of proteins and acted as a bias. Finally, the correlation between blood biomarkers and brain tissue changes was significant only when the whole population was included, whereas the significance disappeared when only patients with cognitive dysfunction were analyzed. Therefore, further studies should be performed to evaluate the correlation in a larger population.

## 4. Materials and Methods

### 4.1. Participants

Our institutional review board approved this prospective study (KHNMC IRB 2009-056 and KHNMC GRRB 2009-004; KHNMC IRB 2011-059 and KHNMC GRRB 2011-008) and informed consent was obtained from all participants. All participants provided a detailed medical history and underwent a neurological examination, standard neuropsychological testing, blood sampling, and MRI. All participants were prospectively recruited in the neurological center of our institution. Cognitive function was assessed using the full Seoul Neuropsychological Screening Battery including K-MMSE, which is a standardized neuropsychological battery in Korea that comprises tests for attention, visuospatial function, verbal and visual memory function, language-related function, and frontal executive function [38]. Neuroradiologists (SP and CWR) with 15 years of imaging experience evaluated brain MRI to determine if there was any evidence of prior cortical infarctions or other space-occupying lesions that would exclude the participant.

Based on the results of neuropsychological tests and MRI findings, we subdivided participants into three groups. First, CN elderly participants were selected from healthy volunteers who did not have a medical history of neurological disease, who showed normal results on detailed cognitive testing scores that were within 1 standard deviation (SD) adjusted for age, sex, and education according to the Korean normative database. Second, patients with a mild-to-moderate degree of probable AD were included, and these patients were defined as those with a Clinical Dementia Rating score of 0.5, 1, or 2, according to the National Institute of Neurological and Communicative Disorders and Stroke-Alzheimer Disease and Related Disorders Association criteria [38,39]. Finally, participants with aMCI were included according to the Petersen criteria [40,41]. Participants who had both MRI data and blood samples were included in this study. Patients with vascular dementia were excluded from this study. Finally, we included 45 CN, 34 aMCI, and 39 AD subjects, 118 participants in total. Table 1 summarizes the demographic characteristics and the results of neuropsychological tests of the three participant groups.

### 4.2. Plasma Levels of HO-1, CypA, and IRE1

This analysis was performed at the lab of one of the co-authors (WC). Target protein levels were obtained according to the manufacturer′s recommended protocol (Elabscience, Houston, TX, USA). Briefly, 100 μL of serially titrated standards and 10 times diluted plasma samples were added to the wells of each antibody precoated micro-well plate. The plates were incubated at 37 °C for 90 min. After incubation, 100 μL of biotinylated detector antibody was added and incubated at 37 °C for 1 h. Then, 100 μL of horseradish peroxidase conjugated working solution was added and incubated for 1 h. After washing with washing buffer, 90 μL of substrate solution was added and incubated for 30 min, then 50 μL of stop solution was added and the absorbance was measured at 450 nm. The concentrations were measured from plasma.

### 4.3. MRI Acquisition

To evaluate brain tissue volumes, a sagittal structural 3D T1-weighted image was acquired with the turbo field-echo sequence, which is similar to the magnetization-prepared rapid acquisition of gradient echo sequence with the following parameters: repetition time = 8.1 ms, echo time = 3.7 ms, flip angle = 8°, field-of-view = 236 × 236 mm^2^, and voxel size = 1 × 1 × 1 mm^3^. In addition, T2-weighted turbo-spin-echo and fluid-attenuated inversion recovery images were acquired to rule out any malformations of the brain. MRI was acquired from each participant using a 3-T MRI system (Achieva, Philips Medical Systems, Best, The Netherlands) equipped with an eight-channel sensitivity encoding head coil.

### 4.4. Imaging Processing

The following post processing steps were performed using the Statistical Parametric Mapping Version 12 (SPM12) program (Wellcome Department of Imaging Neuroscience, University College, London, UK). All 3D T1W images were segmented into gray matter, white matter, and CSF and spatially normalized into a dementia template and brain tissue atlas created in our lab using the computational anatomy toolbox segmentation tool [42]. Both spatially normalized GMV and WMV were smoothed using a Gaussian kernel of 8 × 8 × 8 mm^3^ full width at half-maximum for the following statistical analyses.

### 4.5. Statistical Analyses

#### 4.5.1. Demographic Characteristics, Results of Neuropsychological Tests, and Blood Biomarkers

First, an analysis of covariance test was used for continuous variables to compare demographic characteristics, results of neuropsychological tests, and levels of the blood-borne biomarkers among the three subject groups. If any significant differences were found, then the post hoc test was performed using the Scheffe method. Sex was tested using the chi-squared test. Second, we performed correlation analysis to investigate the relationships of subject demographic characteristics such as age, years of education, MMSE scores, and global segmented brain tissue volumes with levels of the three serum proteins. Additionally, we used partial correlation analyses to assess the relationship of MMSE scores with levels of the three serum proteins after adjusting for age. Furthermore, we also evaluated the relationships among the levels of the three blood biomarkers. For those analyses, α < 0.05 was used to determine significance. The statistical evaluation was performed using the statistical program (MedCalc Software, Acacialaan, Ostend, Belgium).

#### 4.5.2. Voxel-Based Multiple Regression Analyses

To assess the relationship between brain tissue volume changes and the levels of the three blood biomarkers, voxel-based multiple regression analysis was performed using all participant data. In this analysis, the brain tissue volume of each voxel was the dependent variable, while blood levels of CypA, HO-1, or IRE1 were independent variables within the framework of a general linear model that was adjusted for the subject′s age, TIV, sex, and years of education. A significance level of α = 0.0005 was applied without correction for multiple comparisons with a minimum cluster size of at least 100 contiguous voxels. We did not use any multiple comparison methods because the relationships among brain tissue volume change and blood biomarkers were unclear and these analyses were performed to define some ROI areas.

#### 4.5.3. ROI-Based Correlation Analyses

ROI-based analysis was performed to determine correlations between brain tissue volumes at specific areas and levels of plasma biomarkers. ROI areas in GM and WM were defined as regions associated negatively or positively with levels of plasma biomarkers in each measure during voxel-based multiple regression analyses as well as brain areas based on an atlas. Each ROI area was defined as a cluster of significant relationships of GMV or WMV with levels of plasma biomarkers using Marsbar software (Matthew Brett, http://marsbar.sourceforge.net, accessed on 20 April 2021). The first three ROI areas were defined from the results of voxel-based multiple regression analyses among GMV and levels of the three blood-based biomarkers (Supplementary Table S1). The other two ROI areas were defined from voxel-based multiple regression analyses among WMV and levels of the three blood-based biomarkers (Supplementary Table S2). We also defined ROI areas from atlas-based ROI areas, which were three AD signature regions including hippocampus, posterior cingulate, and precuneus in wfu_pickatlas software (http://fmri.wfubmc.edu/software/PickAtlas, accessed on 20 April 2021). The means of both GMV and WMV values for each ROI was extracted from all subjects using Marsbar software. To investigate the relationship of GMV or WMV at each ROI with blood levels of CypA, HO-1, and IRE1, we performed partial correlation analysis, adjusting for age.

## 5. Conclusions

In this study, we demonstrated that blood levels of CypA, HO-1, and IRE1 had a positive (or negative) association with the GMV of signature AD regions, cognitive function, and age. However, it is important to note that this correlation was significant only when the whole population, CN included, was considered, whereas the significance disappeared when only patients with cognitive dysfunction were included. Considering the potential roles of each protein in the pathogenesis of AD, the blood levels of these proteins may reflect pathological mechanisms of AD in the brain and could be candidate blood-borne biomarkers. Further studies are needed to validate our results in much larger populations with AD.

## Figures and Tables

**Table 1:** Summary of demographic data, neuropsychological test results, global brain tissue volume, levels of the three blood-based biomarkers, and statistical analyses.

Group	CN (1)	aMCI (2)	AD (3)	Statistics (Post Hoc)
Demographic Data and Neuropsychologic Tests				
No. of subjects	45	34	39	118
* Age (years)	63.64 ± 9.18	70.35 ± 6.88	75.03 ± 7.92	F = 20.697/*p* < 0.001 (1,2)(1,3)
Sex (male/female)	12/33	6/28	4/35	^&^ 0.1541, χ^2^ = 3.741
* K-MMSE(/30)	27.73 ± 2.32	24.12 ± 4.00	18.49 ± 4.60	F = 65.904/*p* < 0.001 (1,2,3)
CDR (range)	0.000 (0.0–0.5)	0.500 (0.5–0.5)	1.000 (0.50–2.0)	N/A
* Education (years)	9.53 ± 5.98	8.48 ± 5.11	6.26 ± 4.65	F = 4.051/*p* = 0.020 (1,3)
* Global Brain Tissue Segmented Volume				
* Global GMV (mm^3^)	586.36 ± 48.07	564.48 ± 42.50	525.41 ± 52.26	F = 17.053/*p* < 0.001 (1,3) (2,3)
* Global WMV (mm^3^)	471.59 ± 50.50	456.32 ± 39.88	436.34 ± 46.22	F = 6.080/*p* = 0.003 (1,3)
* Global CSF volume (mm^3^)	365.61 ± 54.77	419.54 ± 62.10	486.51 ± 69.61	F = 39.614/*p* < 0.001 (1,2,3)
* TIV (mm^3^)	1423.56 ± 101.07	1440.34 ± 108.99	1448.25 ± 134.34	F = 0.505/*p* = 0.605
* Plasma Levels of Three Blood Biomarkers				
* CypA (PPIA)ng/mL)	1470.44 ± 201.27	1372.13 ± 250.96	1352.80 ± 250.35	F = 3.089/*p* = 0.049 (none)
* HO-1 (ng/mL)	285.05 ± 70.71	280.87 ± 61.25	250.91 ± 56.61	F = 3.415/*p* = 0.036 (none)
* IRE1(pg/mL)	31.30 ± 6.04	30.62 ± 7.01	34.01 ± 6.19	F = 2.995/*p* = 0.054

* *p*-value by analysis of covariance (ANCOVA) with Scheffe post hoc test. ^&^ Gender was compared between the two groups by Chi-square test. Data are listed as arithmetic mean ± standard deviation, except sex and CDR. CDR scores are presented as median (range). CN, cognitive normal; aMCI, amnestic mild cognitive impairment; AD, Alzheimer’s disease; K-MMSE, Korean version of the Mini-Mental State Examination Score; CDR, Clinical Dementia Rating; GMV, gray matter volume; WMV, white matter volume; CSF, cerebrospinal fluid; TIV, total intracranial volume; CypA, cyclophilin A; HO-1, heme oxygenase-1; IRE1, inositol-requiring enzyme 1.

**Table 2:** Correlation analysis between levels of the three blood-based biomarkers, demographic characteristics, and global segmented brain tissue volumes.

Regressor	Subjects	CypA	HO-1	IRE1
Age	All	−0.096/0.303	*−0.226*/*0.014*	*0.222*/*0.016*
	CN	−0.118/0.439	−0.126/0.409	0.011/0.944
	aMCI	−0.215/0.222	*−0.403*/*0.018*	*0.482*/*0.004*
	AD	0.349/0.029	0.042/0.800	0.118/0.474
MMSE	All	*0.215*/*0.019*	*0.250*/*0.006*	*−0.241*/*0.009*
	CN	0.098/0.523	0.009/0.952	0.049/0.7515
	aMCI	0.126/0.478	−0.194/0.272	−0.091/0.607
	AD	0.088/0.594	*0.493*/*0.001*	*−0.344*/*0.032*
* adjMMSE	All	*0.195*/*0.036*	0.160/0.085	−0.153/0.101
	CN	0.054/0.730	−0.048/0.756	0.0584/0.707
	aMCI	0.072/0.689	−0.343/0.051	0.0456/0.801
	AD	0.152/0.363	*0.506*/*0.001*	*−0.332*/*0.042*
Education	All	0.012/0.901	0.092/0.325	−0.038/0.684
TIV	All	0.048/0.609	−0.063/0.498	−0.006/0.947
Global GMV	All	*0.220*/*0.017*	*0.193*/*0.037*	*−0.213*/*0.021*
	CN	0.156/0.305	0.043/0.777	−0.080/0.604
	aMCI	0.182/0.304	0.091/0.607	−0.165/0.353
	AD	0.114/0.489	0.174/0.288	−0.192/0.241
Global WMV	All	0.154/0.097	0.059/0.530	−0.100/0.282
CypA	All	-	0.131/0.156	−0.106/0.252
HO-1	All	0.131/0.156	-	*−0.664*/*<0.001*
	CN	0.145/0.343	-	*−0.627*/*<0.001*
	aMCI	0.060/0.737	-	*−0.756*/*<0.001*
	AD	0.082/0.620	-	*−0.583*/*0.001*

Data are listed as Pearson correlation coefficient *r*/*p*-values, except adjMMSE*. Italics indicate a significant correlation. * adjMMSE: partial correlation analyses between MMSE score and levels of the three plasma biomarkers after adjusting for age. CypA, cyclophilin A; HO-1, heme oxygenase-1; IRE1, inositol-requiring enzyme 1; MMSE, Mini-Mental State Examination Score; GMV, gray matter volume; WMV, white matter volume; TIV, total intracranial volume; CN, cognitive normal; aMCI, amnestic mild cognitive impairment; AD, Alzheimer’s disease.

**Table 3:** Partial correlation analyses between brain tissue volumes and levels of the three plasma biomarkers after adjusting for age of all participants at each region of interest (ROI).

ROI	Tissue	CypA *r*/*p-*Value		HO-1 *r*/*p-*Value		IRE1 *r*/*p-*Value	
Cluster 1	GMV	0.387	*<0.0001*	−0.043	0.642	−0.025	0.789
	WMV	0.054	0.564	0.079	0.394	−0.083	0.372
Cluster 2	GMV	−0.023	0.805	*0.307*	*0.0008*	*−0.239*	*0.010*
	WMV	0.012	0.898	0.013	0.886	−0.065	0.489
Cluster 3	GMV	0.080	0.391	*0.287*	*0.002*	*−0.335*	*0.0002*
	WMV	0.054	0.567	−0.045	0.629	0.027	0.772
Cluster 4	GMV	*0.223*	*0.016*	−0.024	0.801	0.017	0.856
	WMV	*0.369*	*<0.0001*	0.058	0.537	−0.092	0.323
Cluster 5	GMV	0.129	0.165	−0.058	0.532	−0.137	0.140
	WMV	0.655	0.483	*0.225*	*0.015*	*−0.405*	*<0.0001*
Hippocampus	GMV	0.156	0.093	*0.197*	*0.034*	−0.175	0.059
	WMV	0.106	0.256	−0.042	0.650	0.015	0.869
Posterior Cingulate	GMV	*0.196*	*0.034*	−0.007	0.937	−0.072	0.442
	WMV	0.158	0.089	0.014	0.880	−0.061	0.513
Precuneus	GMV	0.148	0.112	−0.070	0.451	0.013	0.887
	WMV	0.138	0.137	0.037	0.696	−0.063	0.495

Data are listed as partial correlation coefficient *r* with *p*-value. Italics indicate a significant correlation. GMV, gray matter volume; WMV, white matter volume; CypA, cyclophilin A; HO-1, heme oxygenase-1; IRE1, inositol-requiring enzyme 1.

## Data Availability

The datasets generated and analyzed during the current study are available from the corresponding author on reasonable request.

## Supplementary Materials

The following are available online at https://www.mdpi.com/article/10.3390/diagnostics11050740/s1. Table S1: Locations of the voxel-based multiple regression analyses between gray matter volume (GMV) and the three blood-based biomarkers in all participants. Table S2: Locations of the voxel-based multiple regression analyses between white matter volume (WMV) and the three blood-based biomarkers in all subjects.

## References

[1] Makin S. (2018). The amyloid hypothesis on trial. Nature.

[2] Shah Y., Tangalos E.G., Petersen R.C. (2000). Mild cognitive impairment. When is it a precursor to Alzheimer’s disease?. Geriatrics.

[3] Apostolova L.G., Hwang K.S., Avila D., Elashoff D., Kohannim O., Teng E., Sokolow S., Jack C.R., Jagust W.J., Shaw L. (2015). Brain amyloidosis ascertainment from cognitive, imaging, and peripheral blood protein measures. Neurology.

[4] Youn Y.C., Kang S., Suh J., Park Y.H., Kang M.J., Pyun J.M., Choi S.H., Jeong J.H., Park K.W., Lee H.W. (2019). Blood amyloid-β oligomerization associated with neurodegeneration of Alzheimer’s disease. Alzheimer’s Res. Ther..

[5] Endres K., Reinhardt S. (2013). ER-stress in Alzheimer’s disease: Turning the scale?. Am. J. Neurodegener Dis..

[6] Satoh K., Shimokawa H., Preedy V.R., Patel V.B. (2015). Cyclophilin A: Novel Biomarker for Oxidative Stress and Cardiovascular Diseases. General Methods in Biomarker Research and their Applications.

[7] Zhao Y., Zhao B. (2013). Oxidative stress and the pathogenesis of Alzheimer’s disease. Oxid. Med Cell Longev..

[8] Bell R.D., Winkler E.A., Singh I., Sagare A.P., Deane R., Wu Z., Holtzman D.M., Betsholtz C., Armulik A., Sallstrom J. (2012). Apolipoprotein E controls cerebrovascular integrity via cyclophilin A. Nature.

[9] Sweeney M.D., Sagare A.P., Zlokovic B.V. (2018). Blood-brain barrier breakdown in Alzheimer disease and other neurodegenerative disorders. Nat. Rev. Neurol..

[10] Smith M.A., Kutty R.K., Richey P.L., Yan S.D., Stern D., Chader G.J., Wiggert B., Petersen R.B., Perry G. (1994). Heme oxygenase-1 is associated with the neurofibrillary pathology of Alzheimer’s disease. Am. J. Pathol..

[11] Hettiarachchi N., Dallas M., Al-Owais M., Griffiths H., Hooper N., Scragg J., Boyle J., Peers C. (2014). Heme oxygenase-1 protects against Alzheimer’s amyloid-β1-42-induced toxicity via carbon monoxide production. Cell Death Dis..

[12] Ryter S.W., Choi A.M. (2016). Targeting heme oxygenase-1 and carbon monoxide for therapeutic modulation of inflammation. Transl. Res. J. Lab. Clin. Med..

[13] Cohen N., Breker M., Bakunts A., Pesek K., Chas A., Argemí J., Orsi A., Gal L., Chuartzman S., Wigelman Y. (2017). Iron affects Ire1 clustering propensity and the amplitude of endoplasmic reticulum stress signaling. J. Cell Sci..

[14] Healy S., McMahon J., FitzGerald U. (2018). UPR Induction Prevents Iron Accumulation and Oligodendrocyte Loss in ex vivo Cultured Hippocampal Slices. Front. Neurosci..

[15] Jack C.R., Barkhof F., Bernstein M.A., Cantillon M., Cole P.E., Decarli C., Dubois B., Duchesne S., Fox N.C., Frisoni G.B. (2011). Steps to standardization and validation of hippocampal volumetry as a biomarker in clinical trials and diagnostic criterion for Alzheimer’s disease. Alzheimer’s Dement. J. Alzheimer’s Assoc..

[16] Leow A.D., Yanovsky I., Parikshak N., Hua X., Lee S., Toga A.W., Jack C.R., Bernstein M.A., Britson P.J., Gunter J.L. (2009). Alzheimer’s disease neuroimaging initiative: A one-year follow up study using tensor-based morphometry correlating degenerative rates, biomarkers and cognition. Neuroimage.

[17] Plácido A.I., Pereira C.M.F., Duarte A.I., Candeias E., Correia S.C., Santos R.X., Carvalho C., Cardoso S., Oliveira C.R., Moreira P.I. (2014). The role of endoplasmic reticulum in amyloid precursor protein processing and trafficking: Implications for Alzheimer’s disease. Biochim. Biophys. Acta (BBA) Mol. Basis Dis..

[18] Femminella G.D., Dani M., Wood M., Fan Z., Calsolaro V., Atkinson R., Edginton T., Hinz R., Brooks D.J., Edison P. (2019). Microglial activation in early Alzheimer trajectory is associated with higher gray matter volume. Neurology.

[19] Montagne A., Nation D.A., Sagare A.P., Barisano G., Sweeney M.D., Chakhoyan A., Pachicano M., Joe E., Nelson A.R., D’Orazio L.M. (2020). APOE4 leads to blood-brain barrier dysfunction predicting cognitive decline. Nature.

[20] Bejanin A., Schonhaut D.R., La Joie R., Kramer J.H., Baker S.L., Sosa N., Ayakta N., Cantwell A., Janabi M., Lauriola M. (2017). Tau pathology and neurodegeneration contribute to cognitive impairment in Alzheimer’s disease. Brain A J. Neurol..

[21] Dickerson B.C., Stoub T.R., Shah R.C., Sperling R.A., Killiany R.J., Albert M.S., Hyman B.T., Blacker D., Detoledo-Morrell L. (2011). Alzheimer-signature MRI biomarker predicts AD dementia in cognitively normal adults. Neurology.

[22] Hirose W., Ikematsu K., Tsuda R. (2003). Age-associated increases in heme oxygenase-1 and ferritin immunoreactivity in the autopsied brain. Leg. Med..

[23] Schipper H.M. (2011). Heme oxygenase-1 in Alzheimer disease: A tribute to Moussa Youdim. J. Neural Transm..

[24] Di Domenico F., Barone E., Mancuso C., Perluigi M., Cocciolo A., Mecocci P., Butterfield D.A., Coccia R. (2012). HO-1/BVR-A System Analysis in Plasma from Probable Alzheimer’s Disease and Mild Cognitive Impairment Subjects: A Potential Biochemical Marker for the Prediction of the Disease. J. Alzheimer’s Dis..

[25] Hampel H., Bürger K., Teipel S.J., Bokde A.L.W., Zetterberg H., Blennow K. (2008). Core candidate neurochemical and imaging biomarkers of Alzheimer’s disease⁎⁎This paper was presented in part by the 1st author at the 10th International Conference of Alzheimer’s Disease and Related Disorders (ICAD), Madrid, Spain, July 2006, as an invited plenary lecture. Alzheimer’s Dement..

[26] Maes O.C., Kravitz S., Mawal Y., Su H., Liberman A., Mehindate K., Berlin D., Sahlas D.J., Chertkow H.M., Bergman H. (2006). Characterization of alpha1-antitrypsin as a heme oxygenase-1 suppressor in Alzheimer plasma. Neurobiol. Dis..

[27] Kimpara T., Takeda A., Watanabe K., Itoyama Y., Ikawa S., Watanabe M., Arai H., Sasaki H., Higuchi S., Okita N. (1997). Microsatellite polymorphism in the human heme oxygenase-1 gene promoter and its application in association studies with Alzheimer and Parkinson disease. Hum. Genet..

[28] Shibata N., Ohnuma T., Baba H., Arai H. (2009). No genetic association between polymorphisms of heme oxygenase 1 and 2 and Alzheimer’s disease in a Japanese population. Dement Geriatr. Cogn. Disord..

[29] Eastman J.A., Hwang K.S., Lazaris A., Chow N., Ramirez L., Babakchanian S., Woo E., Thompson P.M., Apostolova L.G. (2013). Cortical thickness and semantic fluency in Alzheimer’s disease and mild cognitive impairment. Am. J. Alzheimers Dis..

[30] de Jong L.W., van der Hiele K., Veer I.M., Houwing J.J., Westendorp R.G., Bollen E.L., de Bruin P.W., Middelkoop H.A., van Buchem M.A., van der Grond J. (2008). Strongly reduced volumes of putamen and thalamus in Alzheimer’s disease: An MRI study. Brain A J. Neurol..

[31] Haroutunian V., Katsel P., Schmeidler J. (2009). Transcriptional vulnerability of brain regions in Alzheimer’s disease and dementia. Neurobiol. Aging.

[32] Cornejo V.H., Hetz C. (2013). The unfolded protein response in Alzheimer’s disease. Semin. Immunopathol..

[33] Duran-Aniotz C., Cornejo V.H., Espinoza S., Ardiles Á.O., Medinas D.B., Salazar C., Foley A., Gajardo I., Thielen P., Iwawaki T. (2017). IRE1 signaling exacerbates Alzheimer’s disease pathogenesis. Acta Neuropathol..

[34] Li J., Pan P., Huang R., Shang H. (2012). A meta-analysis of voxel-based morphometry studies of white matter volume alterations in Alzheimer’s disease. Neurosci. Biobehav. Rev..

[35] Guo X., Wang Z., Li K., Li Z., Qi Z., Jin Z., Yao L., Chen K. (2010). Voxel-based assessment of gray and white matter volumes in Alzheimer’s disease. Neurosci. Lett..

[36] Drummond G.S., Baum J., Greenberg M., Lewis D., Abraham N.G. (2019). HO-1 overexpression and underexpression: Clinical implications. Arch. Biochem. Biophys..

[37] Nigro P., Pompilio G., Capogrossi M.C. (2013). Cyclophilin A: A key player for human disease. Cell Death Dis..

[38] Ahn H.J., Chin J., Park A., Lee B.H., Suh M.K., Seo S.W., Na D.L. (2010). Seoul Neuropsychological Screening Battery-dementia version (SNSB-D): A useful tool for assessing and monitoring cognitive impairments in dementia patients. J. Korean Med. Sci..

[39] McKhann G., Drachman D., Folstein M., Katzman R., Price D., Stadlan E.M. (1984). Clinical diagnosis of Alzheimer’s disease: Report of the NINCDS-ADRDA Work Group under the auspices of Department of Health and Human Services Task Force on Alzheimer’s Disease. Neurology.

[40] Petersen R.C., Doody R., Kurz A., Mohs R.C., Morris J.C., Rabins P.V., Ritchie K., Rossor M., Thal L., Winblad B. (2001). Current concepts in mild cognitive impairment. Arch. Neurol..

[41] Petersen R.C., Smith G.E., Waring S.C., Ivnik R.J., Tangalos E.G., Kokmen E. (1999). Mild cognitive impairment: Clinical characterization and outcome. Arch. Neurol..

[42] Seiger R., Ganger S., Kranz G.S., Hahn A., Lanzenberger R. (2018). Cortical Thickness Estimations of FreeSurfer and the CAT12 Toolbox in Patients with Alzheimer’s Disease and Healthy Controls. J. Neuroimaging.

