# Supplementary material for: Relationship between Brain Tissue Changes and Blood Biomarkers of Cyclophilin A, Heme Oxygenase-1, and Inositol-Requiring Enzyme 1 in Patients with Alzheimer’s Disease
Source: Diagnostics (Basel). 2021 Apr 21;11(5):740. doi: 10.3390/diagnostics11050740 (PMC8143350; doi:10.3390/diagnostics11050740)
Supplement: Supplementary file 1 [file diagnostics-11-00740-s001.zip › diagnostics-1172145-supplementary.pdf]

Supplementary Figures and Tables

Supplementary Table S1. Locations of the voxel-based multiple regression analyses between gray matter volume (GMV) and the three blood-based biomarkers in all participants.

Group Analysis	Cluster Size	Cluster Location	BA	Talairach Coordinates	Z Score	ROI
		**Positive CypA**				
	560	Rt middle occipital gyrus	19	31.99, −80.98, 5.25	4.263	**Cluster1**
		Rt lingual gyrus	18	32.18, −71.29, −7.34	3.844	
	227	Rt middle temporal gyrus	21	55.95, −18.05, −4.59	4.007	
		Rt superior temporal gyrus	22	49.06, −21.81, −9.12	3.919	
	162	Rt middle temporal gyrus	22	48.79, −42.94, 5.09	3.736	
	213	Rt cerebellum, declive		15.65, −71.68, −17.11	3.705	
	112	Rt cerebellum, culmen		24.03, −49.37, −14.86	3.664	
		Rt temporal fusiform gyrus	37	32.39, −43.69, −15.53	3.456	
		**Negative CypA**				
	116	Lt limbic posterior cingulate	23	−2.72, −27.34, 20.56	3.903	
		**Positive HO-1**				
	163	Lt parietal postcentral gyrus	7	−18.67, −48.74, 66.91	4.004	
	154	Lt limbic lobe uncus	28	−31.17, 2.66, −25.73	3.996	**Cluster2**
		**Negative IRE1**				
	176	Lt lateral globus pallidus		−20.37, −14.73, −6.92	3.933	**Cluster3**
		Lt Putamen		−24.54, −5.18, −3.39	3.479	
	101	Rt limbic lobe uncus	28	14.69, 6.88, −27.25	3.878	
	112	Rt hypothalamus		7.52, −1.64, −11.97	3.837	

We adjusted subject's total intracranial volume (TIV), gender, age and education-year as covariates. A significance level of $p = 0.0005$ was applied without correction for multiple comparisons with the minimum cluster size at least 100 contiguous voxels. ROI, region-of-interest; Rt, right; Lt, left; BA, Brodmann area; CypA, cyclophilin A; HO-1, heme oxygenase-1; IRE1, inositol-requiring enzyme 1.

Supplementary Table S2. Locations of the voxel-based multiple regression analyses between white matter volume (WMV) and the three blood-based biomarkers in all subjects.

Group Analysis	Cluster Size	Cluster Location	BA	Talairach Coordinates	Z Score	ROI
		**Positive CypA**				
	104	Rt inferior parietal lobule		46.95, −38.08, 42	4.325	
	247	Rt middle temporal gyrus		53.32, −11.53, −13.48	4.325	**Cluster4**
		**Negative IRE1**				
	237	Lt parietal lobe precuneus	31	−8.51, −72.95, 25.6	4.274	**Cluster5**
		Lt occipital lobe cuneus	18	−1.61, −76.04, 28.13	3.809	
		Rt occipital lobe cuneus	19	6.66, −76.47, 32.28	3.697	
	172	Rt superior parietal lobule	7	28.82, −50.95, 44.53	3.848	

We adjusted subject's total intracranial volume (TIV), age and education-year as covariates. A significance level of p = 0.0005 was applied without correction for multiple comparisons with the minimum cluster size at least 100 contiguous voxels. ROI, region-of-interest; Rt, right; Lt, left; BA, Brodmann area; CypA, cyclophilin A; HO-1, heme oxygenase-1; IRE1, inositol-requiring enzyme 1.

The cluster ROIs are listed in the Supplementary Table 1 and 2: Cluster 1 is the area of the right middle occipital gyrus and lingual gyrus, Cluster 2 is the area of the left uncus in the limbic lobe, Cluster 3 is the area of the left lateral globus pallidus and putamen, Cluster 4 is the area of the right middle temporal gyrus, and Cluster 5 is the area of the left and right cuneus in the occipital lobe and left precuneus in the parietal lobe. Other graphs are shown in the Supplementary Figure 1.

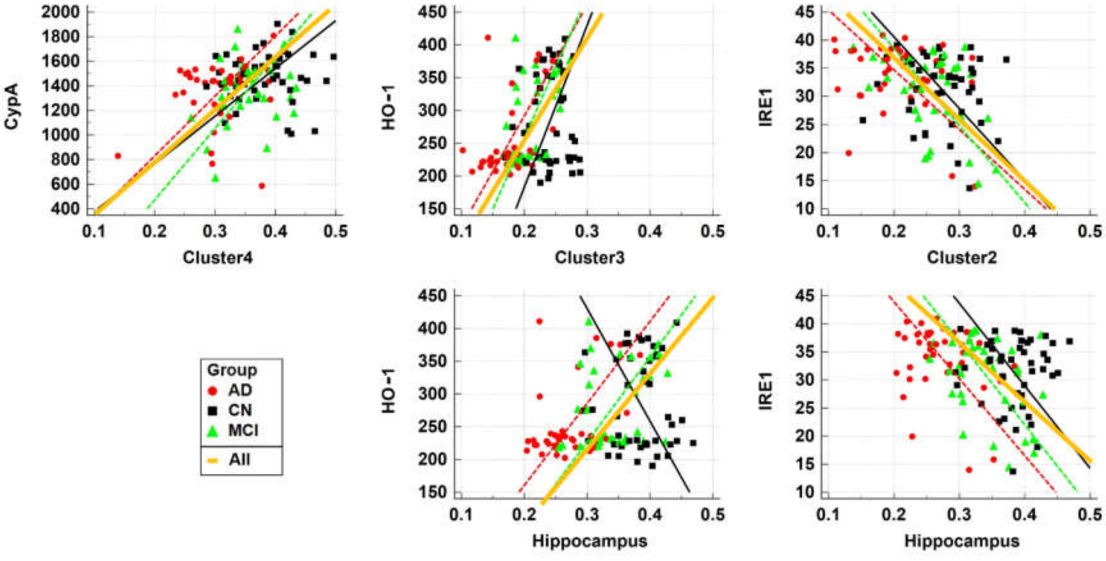

Supplementary Figure S1.
